# Possible Inhibitor from Traditional Chinese Medicine for the ***β*** Form of Calcium-Dependent Protein Kinase Type II in the Treatment of Major Depressive Disorder

**DOI:** 10.1155/2014/761849

**Published:** 2014-06-18

**Authors:** Tzu-Chieh Hung, Wen-Yuan Lee, Kuen-Bao Chen, Hung-Jin Huang, Yueh-Chiu Chan, Cheng-Chun Lee, Calvin Yu-Chian Chen

**Affiliations:** ^1^Department of Biomedical Informatics, Asia University, Taichung 41354, Taiwan; ^2^School of Medicine, College of Medicine, China Medical University, Taichung 40402, Taiwan; ^3^Department of Neurosurgery, China Medical University Hospital, No. 2, Yude Road, North District, Taichung 40447, Taiwan; ^4^Department of Anesthesiology, China Medical University Hospital, Taichung 40447, Taiwan; ^5^Department of Chinese Pharmaceutical Sciences and Chinese Medicine Resources, College of Pharmacy, China Medical University, Taichung 40402, Taiwan; ^6^Research Center for Chinese Medicine & Acupuncture, China Medical University, Taichung 40402, Taiwan; ^7^Human Genetic Center, Department of Medical Research, China Medical University Hospital, Taichung 40447, Taiwan

## Abstract

Recently, an important topic of major depressive disorder (MDD) had been published in 2013. MDD is one of the most prevalent and disabling mental disorders. Consequently, much research is being undertaken into the causes and treatment. It has been found that inhibition of the *β* form of calcium/calmodulin-dependent protein kinase type II (*β*-CaMKII) can ameliorate the disorder. Upon screening the traditional Chinese medicine (TCM) database by molecular docking, sengesterone, labiatic acid, and methyl 3-O-feruloylquinate were selected for molecular dynamics. After 20 ns simulation, the RMSD, total energy, and structure variation could define the protein-ligand interaction. Furthermore, sengesterone, the principle candidate compound, has been found to have an effect on the regulation of emotions and memory development. In structure variation, we find the sample functional group of important amino acids make the protein stable and have limited variation. Due to similarity of structure variations, we suggest that these compounds may have an effect on *β*-CaMKII and that sengesterone may have a similar efficacy as the control. However labiatic acid may be a stronger inhibitor of *β*-CaMKII based on the larger RMSD and variation.

## 1. Introduction

The calcium/calmodulin-dependent protein kinase type II family (CAMKII) has been reported to be related to stress and antidepressant response [1, 2]. However, a recent paper in science [3] indicates that the *β* form of calcium/calmodulin-dependent protein kinase type II (*β*-CaMKII) is related to major depressive disorder (MDD).

MDD is one of the most prevalent and disabling mental disorders. Patients suffering from this disorder are characterized by having a pervasive and persistent low mood that is accompanied by low self-esteem, despair, and may go on to develop anhedonia, which manifests as a loss of interest or pleasure in normally enjoyable activities.

Social pressures associated with the recent economic downturn and work pressure may push more and more people to suffer from this disorder. One of the dangers of this mental disorder is that the patient may indulge in self-harm and may even attack or commit suicide.

From the above description, having a drug to ameliorate the emotional state and treat the disorder is important. According to reference [3], an overexpression of *β*-CaMKII will cause anhedonia. Therefore, MDD could be susceptible to treatment by *β*-CaMKII inhibition.

Computer-aided drug design (CADD) is a rapid and low-cost* in silico* simulation technique to screen for novel drug-candidate compounds by structure and prediction of biological activity. CADD has two major application areas, those of structure-based drug design and ligand-based drug design. The structure-based drug design system contains molecular docking and molecular dynamics simulation [4–9]. These techniques can help in the analysis of protein-ligand interactions by structure variation.

An understanding of personalized medicine and biomedicine, such as the analysis of regional diseases [10], clinical diagnosis cases, and disease associated mutations [11], has recently been attracting more and more attention [12]. Traditional Chinese medicine (TCM) is defined as a personalized medicine that has long been popular in Asia and has an important role in Asian culture. The largest traditional Chinese medicine database in the world, the TCM Database@Taiwan (http://tcm.cmu.edu.tw/) [13], has been established. In this database, 2D chemical structure, 3D chemical structure, and the bioactivity of 61,000 compounds extracted from TCM herbs can be searched. Since 2011, the TCM Database@Taiwan has been investigated for treatments for insomnia [14], pigmentary disorders [15], Parkinson's disease prevention [16], EGFR inhibition [17], pain relief [5], and antivirals [18–22]. The TCM Database@Taiwan can help users to screen TCM compounds via a cloud computing platform [23, 24].

In this research, we look for TCM compounds to inhibit *β*-CaMKII by analyzing their interactions. Candidate compounds are selected according to the docking and structure variations which outperform a control.

## 2. Materials and Methods

### 2.1. Data Set

The Accelrys Discovery Studio 2.5 (DS 2.5) was the platform used for molecule simulation. The TCM compounds were downloaded from TCM database (http://tcm.cmu.edu.tw/). The *β*-CaMKII (PDB ID: 3BHH) crystal structure and the control (4-((4-((5-cyclopropyl-1H-pyrazol-3-yl)amino)-6-(methylamino)pyrimidin-2-yl)amino)phenyl) acetonitrile (also called 5CP and DB07168) were downloaded from the PDB database [25]. The ID of 5CP in PubChem is CID 23624249, and this drug has been defined as a ligand for human calcium/calmodulin-dependent protein kinase IIB isoform 1 (camk2b).

### 2.2. Disorder Protein Detection

Protein disorder will define the difficulty with which a drug affects a protein. For this reason, the protein sequence should be submitted to the database of protein disorder (DisProt, http://www.disprot.org/) for disorder prediction [26].

The important amino acids are defined as Gly21, Val28, Ala41, Lys43, Val74, Phe90, Gly95, Gly96, Gln97, Asn141, Leu143, Asp157, and Phe158 which can interact with 5CP (defined by Lichtarge lab 2006). The disorder prediction can identify the protein character of the docking site, allowing the conditions of the protein-ligand interaction to be discussed.

### 2.3. Molecular Docking

LigandFit [27] is a receptor-rigid docking algorithm program in Discovery Studio 2.5 (DS 2.5) under the force field of CHARMm [28]. The docking site was established as the 5CP docking site, which is near to important amino acids. Ligplot analyzes the hydrophobic interactions for the selected compounds after docking [29, 30].

### 2.4. Molecular Dynamics Simulation

These ligands must be prepared by using SwissParam (http://swissparam.ch/) [31] on the force field [32] of GROMACS 4.5.5 [33]. After ligand preparation, the complex of *β*-CaMKII and ligands are transferred to the buffer (or solution) simulation box. The distance between the complex and the box is a minimum distance of 1.2 Å. This cubic box is a TIP3P water-solution model containing sodium and chloride ions to neutralize complex charges. The minimization used the steepest descent method for 5,000 steps before transferring the final structure to MD simulation. The electrostatic interactions were on the basis of the particle-mesh Ewald (PME) method with 2 fs per time step for a total of 10,000,000 iterations for calculations [34]. The equilibration was based on the Berendsen weak thermal coupling method under a 100 ps constant temperature (NVT ensemble). After 20 ns of simulation, the MD trajectories, RMSD, and energy variations of the complex were analyzed using a series of the protocols in Gromacs.

## 3. Results and Discussion

### 3.1. The Detection of Disorder Protein

**Figure 1:**
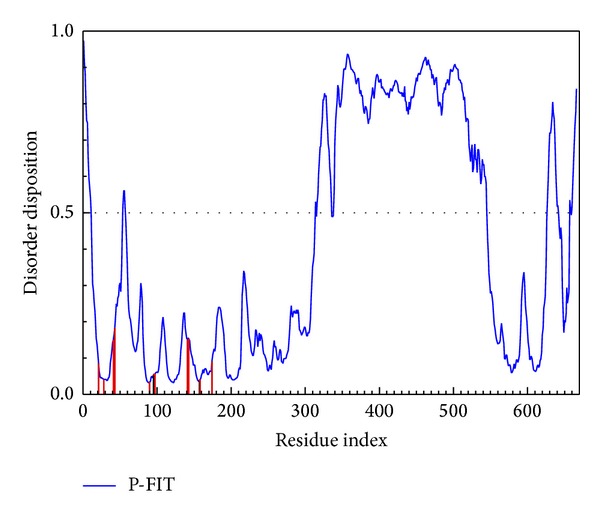
The disorder region prediction and binding site detection. The green curve is the disorder disposition of each amino acid, and the red lines are the residues of the important amino acids.

Protein disorder refers to an unstructured protein, and such characters for the docking site will make drug docking to the protein challenging and thus the complex will not be able to stabilize easily. Recent references [7, 35] indicate that protein disorder is not a common domain; thus, the drug will not dock to similar domains and become responsible for developing side effects. For the above reason, disorder for drug design is not a bad situation and should be defined as difficult work only. The important amino acids of *β*-CaMKII, Gly21, Val28, Ala41, Lys43, Val74, Phe90, Gly95, Gly96, Gln97, Asn141, Leu143, Asp157, and Phe158, are defined as nondisorder regions (Figure 1). For the discussion about the disordered region, the complex with *β*-CaMKII and compounds may be stable.

### 3.2. Molecular Docking

**Figure 2:**
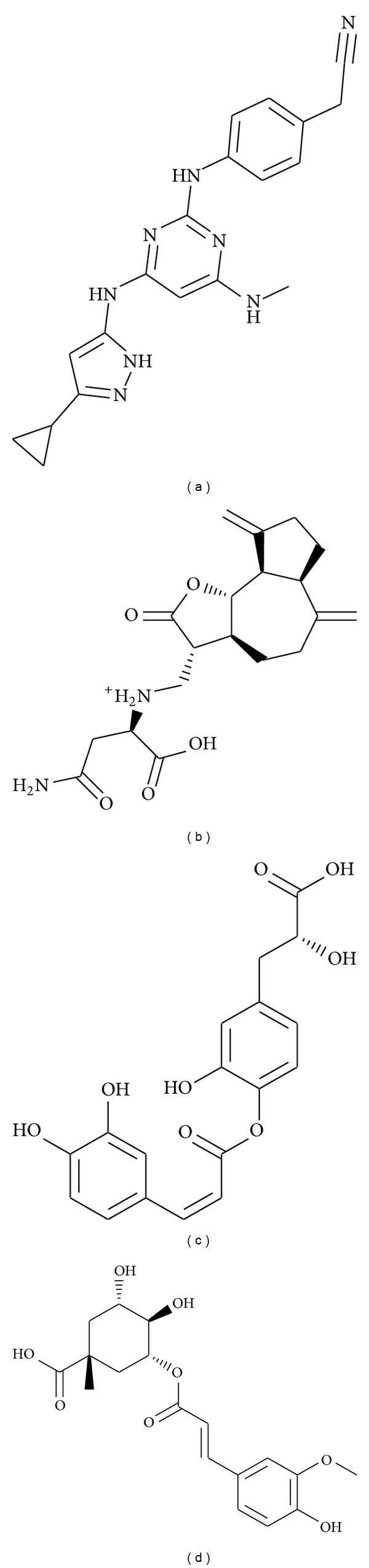
The 2D structure of the control and candidate TCM compounds. (a) 5CP, (b) sengesterone, (c) labiatic acid, and (d) methyl 3-O-feruloylquinate.

The top three TCM compounds from the database were selected as candidate compounds for molecular dynamics, according to the ranked docking score (Table 1). These compounds were in order sengesterone, extracted from* Cyathula capitata* Moq., labiatic acid, extracted from* Rosmarinus officinalis* L., and methyl 3-O-feruloylquinate, extracted from* Phellodendron chinense* C. K. Schneid. (Figure 2). Sengesterone, from the herb* Cyathula capitata,* has been shown to be an insect metamorphosing substance [36]. Labiatic acid from the herb* Rosmarinus officinalis* L. is analgesic and anti-inflammatory [37], has hypoglycemic and hepatoprotective activity [38], regulates glucose and lipid metabolism [39], attenuates oxidative stress, reduces blood cholesterol concentrations [40], improves memory impairment, and affects acetylcholinesterase and butyrylcholinesterase activities [41]. Methyl 3-O-feruloylquinate is an antiviral that has been used in the treatment of H5N1 [42] and, from* Phellodendron amurense* Rupr., can regulate fatty acids [43], protect human osteoarthritic cartilage [44], treat Alzheimer's disease [45], and have an anti-inflammatory [46], an antimicrobial, and anti-herpes simplex virus type 1 activity [47]. This compound has also been found to suppress the cellular immune response [48, 49]. The compounds other than the top compound have been found to regulate memory and emotion [41, 45].

**Figure 3:**
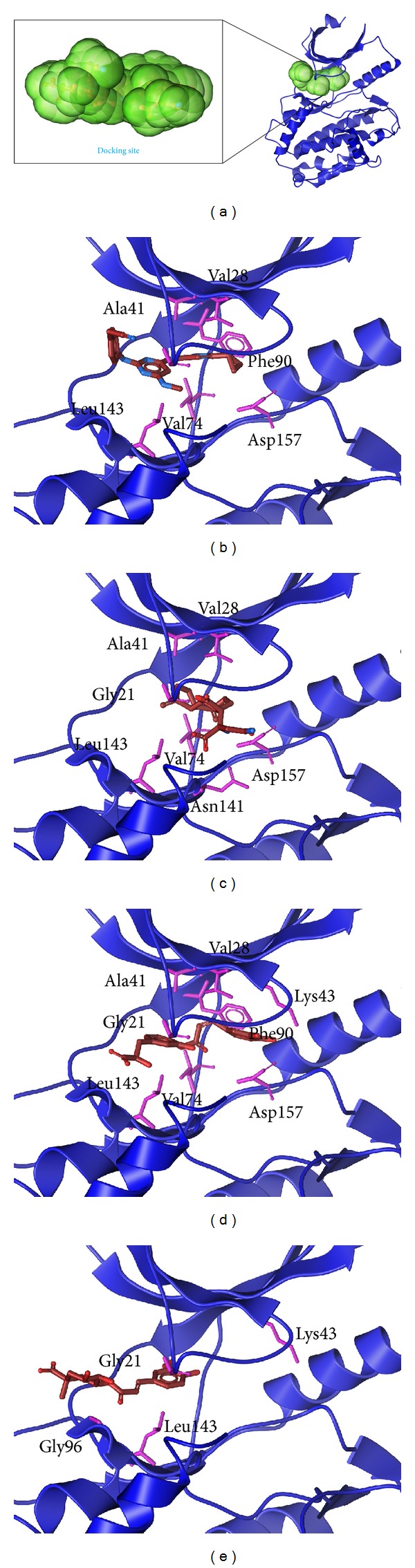
The docking poses of the ligands. (a) The crystal structure of *β*-CaMKII and the docking site, (b) 5CP, (c) sengesterone, (d) labiatic acid, and (e) methyl 3-O-feruloylquinate.

The docking poses are presented in Figure 3, and these results show the ligand interaction with the important amino acids of *β*-CaMKII. From this result, it can be seen that similar amino acids indicate that the ligand may have the same effect while docking.

**Figure 4:**
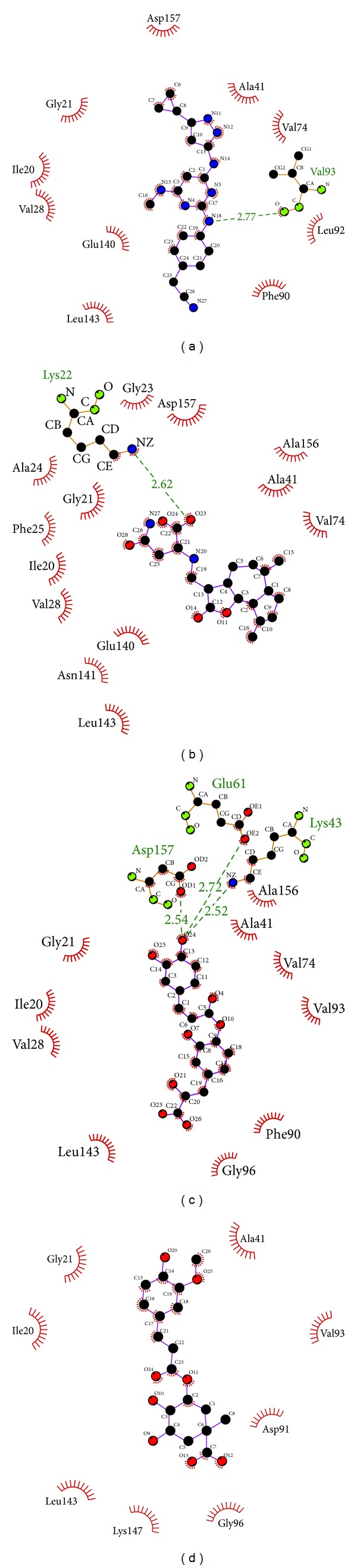
Ligplot illustrates the hydrophobic interactions. (a) 5CP, (b) sengesterone, (c) labiatic acid, and (d) methyl 3-O-feruloylquinate. The deep red color of the hydrophobic interactions indicates a high frequency in all ligand interactions.

The hydrophobic interaction is calculated by Ligplot (Figure 4). The important amino acids Gly21, Val28, Ala41, Lys43, Val74, Phe90, Gly96, Gln97, Leu143, and Asp157 have a high frequency in the result. This indicates that the defined simulation could present the real protein-ligand binding situation.

### 3.3. Molecular Dynamics Simulation

**Figure 5:**
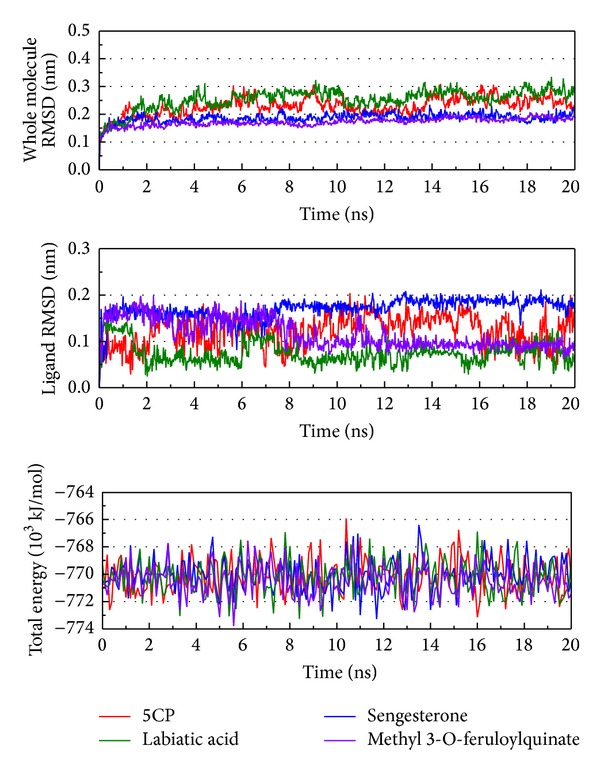
Measures of the MD trajectories. (a) Complex RMSD, (b) ligand RMSD, and (c) the total energy.

The variation of complex RMSD, ligand RMSD, and total energy can help analyze the situation during MD (Figure 5). In Figure 5, the RMSD of the complex and ligand are around, or lower than, 0.2 nm. This result indicates that the protein, ligand, and their complex are stable and that their position and structure variation are not too large. In this ligand, RMSD has a large variation; however, labiatic acid and the complex RMSD of labiatic acid and methyl 3-O-feruloylquinate have a small variation, suggesting that the effect of labiatic acid is moderate. The total energy tends to the range between −772000 and −778000 kJ/mol. From these results, we suggest this simulation will balance quickly according to the stable character of the protein.

**Figure 6:**
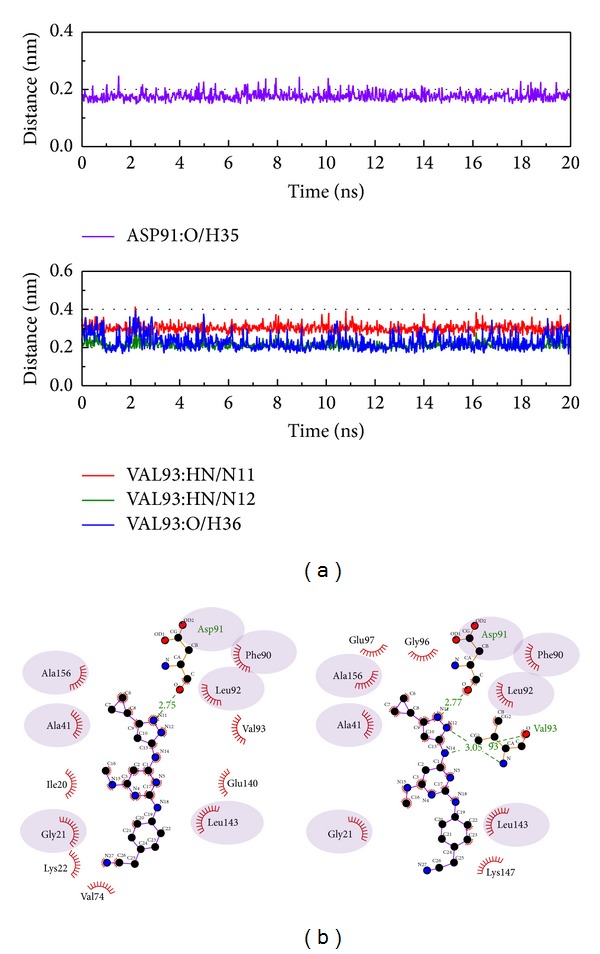
The interaction of 5CP and *β*-CaMKII complex in MD simulation. (a) H-bond variation and (b) hydrophobic variation.

**Figure 7:**
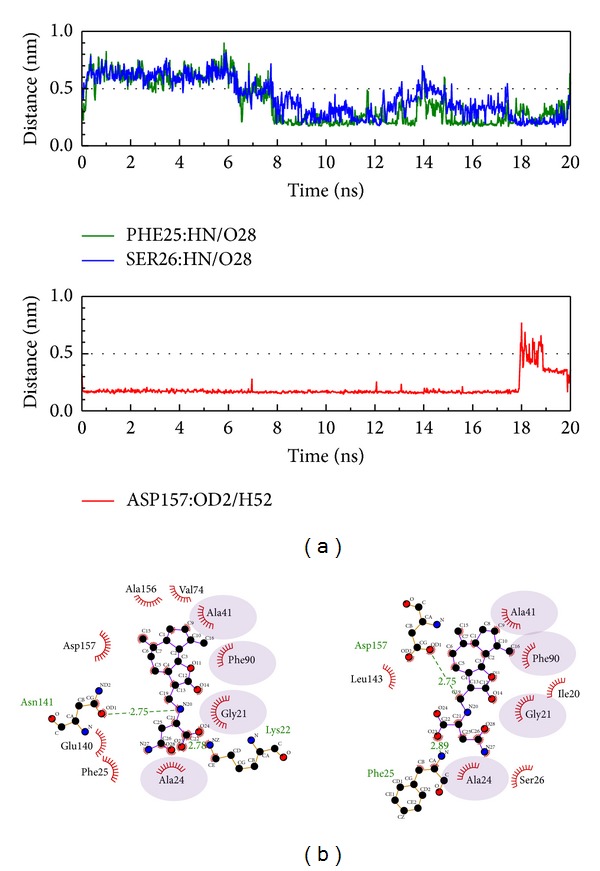
The interaction of sengesterone and *β*-CaMKII complex in MD simulation. (a) H-bond variation and (b) hydrophobic variation.

**Figure 8:**
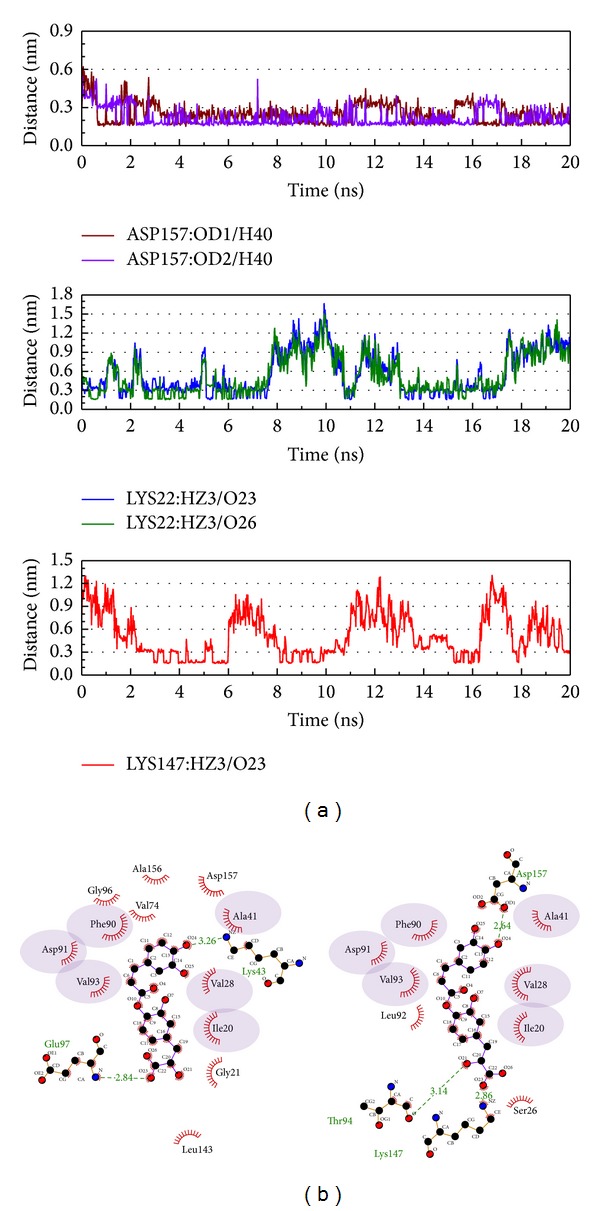
The interaction of labiatic acid and *β*-CaMKII complex in MD simulation. (a) H-bond variation and (b) hydrophobic variation.

**Figure 9:**
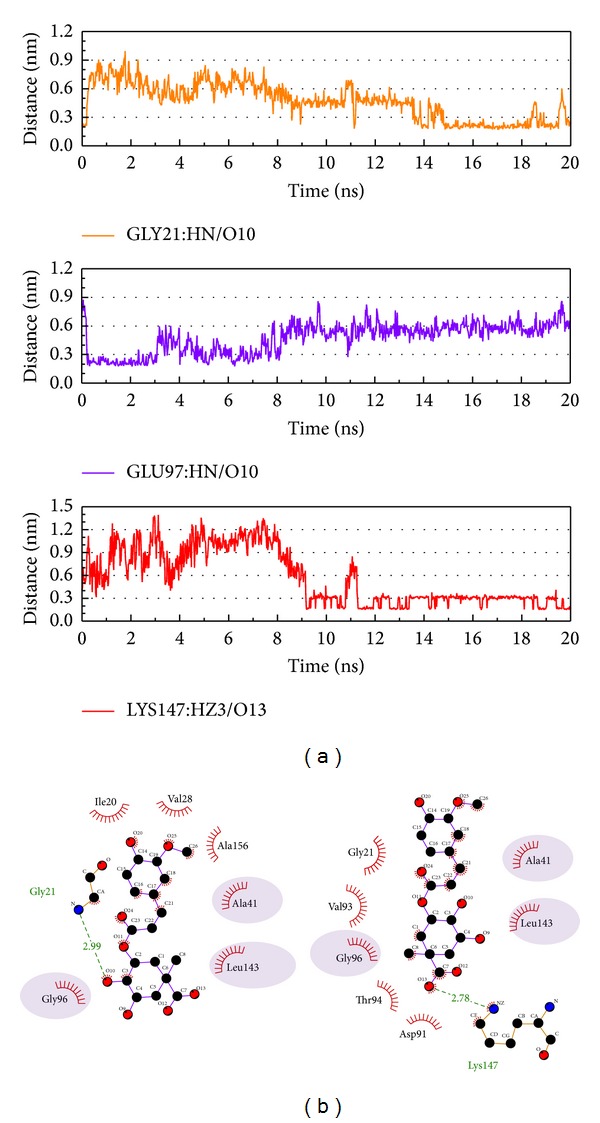
The interaction of methyl 3-O-feruloylquinate and *β*-CaMKII complex in MD simulation. (a) H-bond variation and (b) hydrophobic variation.

Next, we discuss the hydrogen bond variation between protein-control interaction and protein-compounds interaction (Figures 6, 7, 8, and 9). In Figure 6(a), we selected the protein atom with H-bond (defined by the distance <0.3 nm) occupancy greater than 20% to define the interaction and then we compared the different interactions between MD 0 ns and MD 20 ns in Figure 6(b). In this result, it can be found that Asp91 has an important role in CAMKII, as this amino acid maintains the H-bond throughout the MD simulation. Val93 also has high occupancy and has many simultaneous H-bonds from different atoms. As Val93 does not produce H-bonds at the beginning of the MD simulation, we suggest that Val 93 has a greater effect on interactions than the ligand target.

Based on the above description, Asp157 is important in the interaction from the start of the MD simulation as the distance becomes shorter and H-bonds are produced with the principle compound, as seen in Figure 7. After the analysis of the interaction data from the second and third compounds chosen, we could not find one H-bond occupancy over 50%. When we censored the H-bond interactions, we found that these amino acids were all in the domain which influences protein kinase, especially in the region 20–28, which is the ATP and nucleotide binding site. The difference from the control is that the TCM compounds interact with amino acids and become H-bonded in sequences 20–28 and 147–157, but not in sequences 90–98. We suggest the regions other than region 20–28 may be important in *β*-CaMKII.

**Figure 10:**
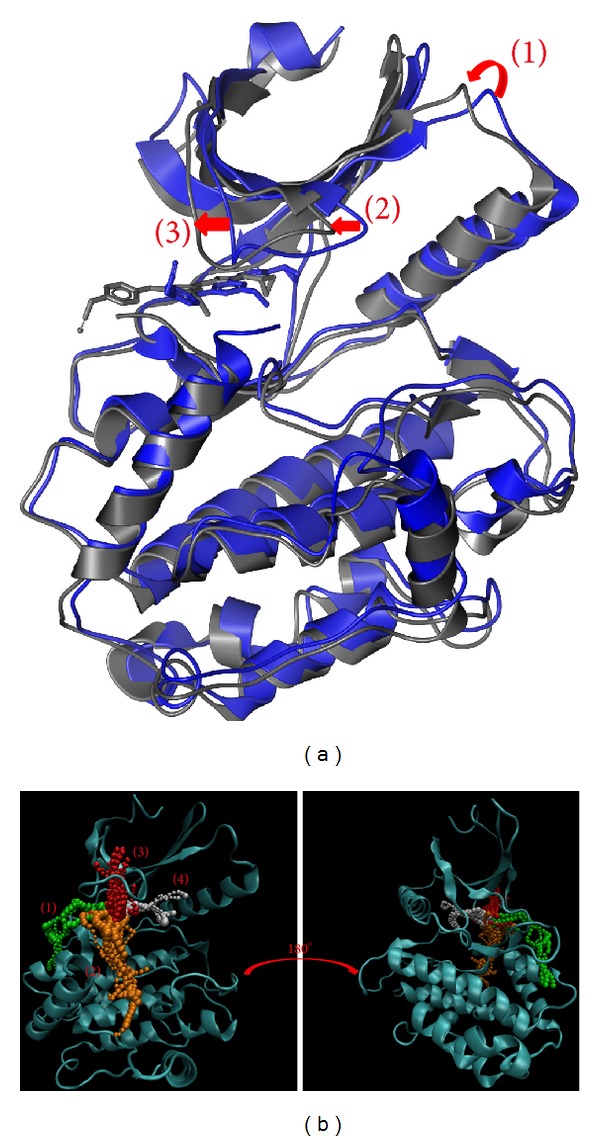
The variation of 5CP and *β*-CaMKII complex in MD simulation. (a) Structure variation and (b) pathway prediction.

**Figure 11:**
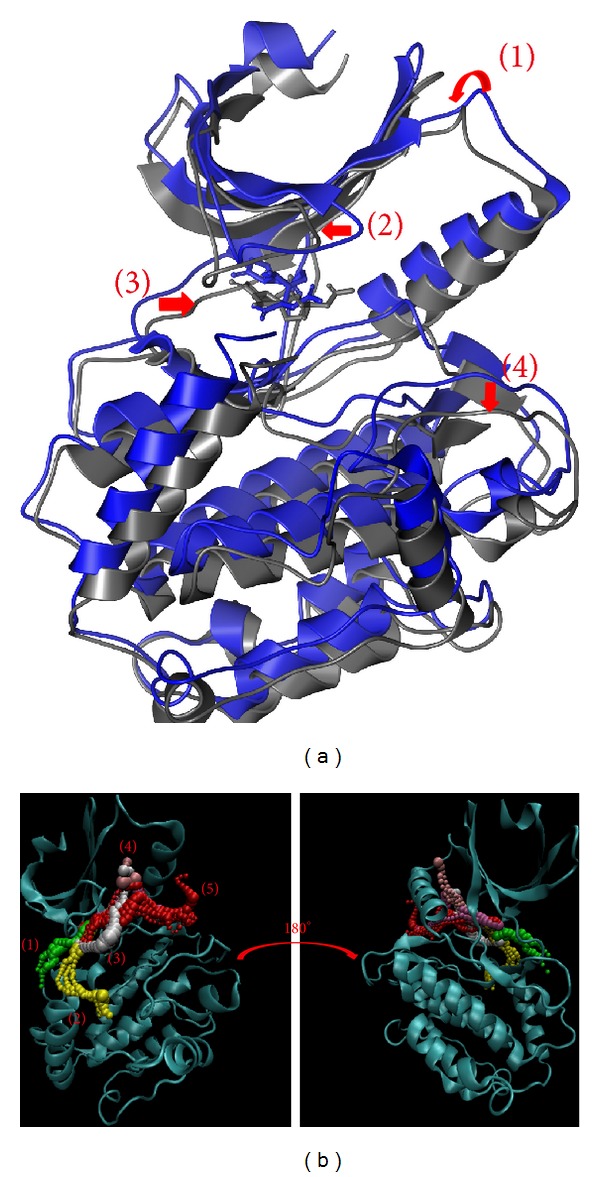
The variation of sengesterone and *β*-CaMKII complex in MD simulation. (a) Structure variation and (b) pathway prediction.

**Figure 12:**
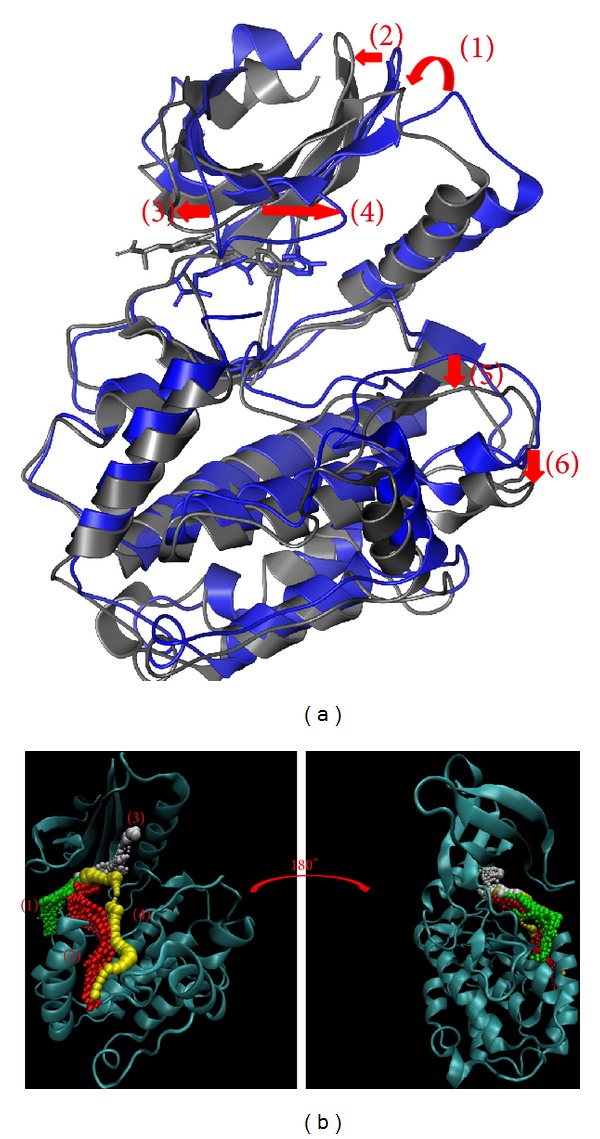
The variation of labiatic acid and *β*-CaMKII complex in MD simulation. (a) Structure variation and (b) pathway prediction.

**Figure 13:**
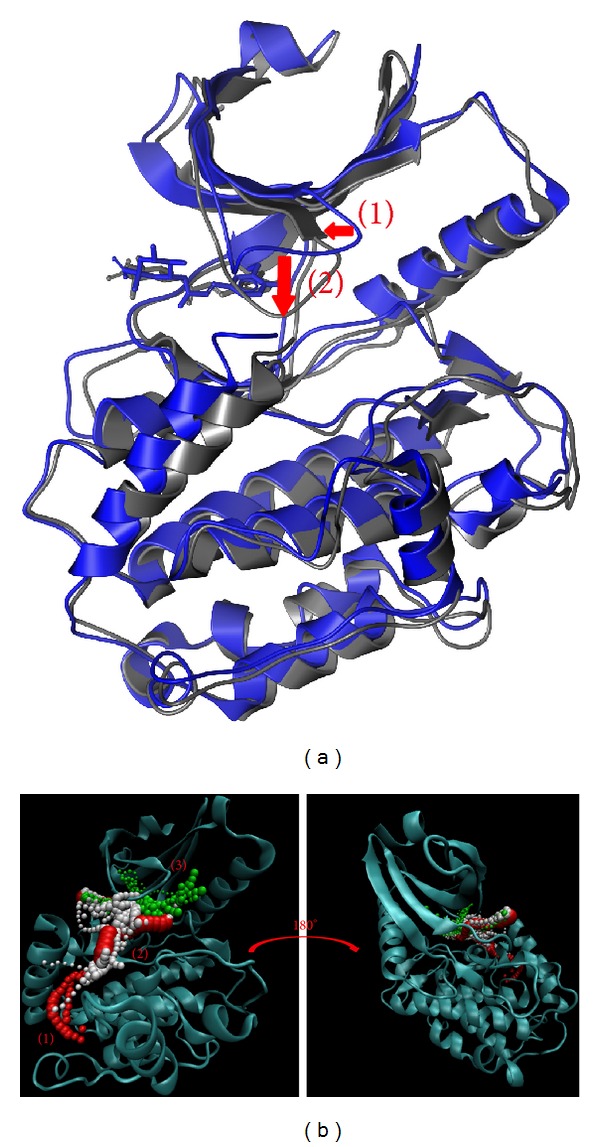
The variation of methyl 3-O-feruloylquinate and *β*-CaMKII complex in MD simulation. (a) Structure variation and (b) pathway prediction.

Finally, we discuss the interaction based on structure variation and ligand pathways in proteins (Figures 10–13). The pathway definition is based on the calculation of caver 3.0 to determine the interpath protein path during MD simulation [50].

In Figure 10(a), there are three large protein variation sites while the ligand moves away during MD simulation. We suggest this variation makes the pore smaller so that no other ligand can have an effect on the domain. The result in Figure 10(b) shows the top four length pathways for 5CP. But, in these pathways, the third is in the protein structure and not in the docking site. In actuality, the ligand could not move through the protein structure even if the path range could allow the ligand to pass. Although there are still three possible pathways, Figure 10(a) shows the ligand moving away from the protein. Thus, ligand 1 or 2 may be the pathway for 5CP. The difference from the control is that sengesterone, as the ligand target, moves inside the protein structure after MD simulation. In Figure 11(a), the structure variations of 1 and 2 are similar, but variation 3 is the opposite. We suggest this interaction causes the pore of the protein to change and thus inhibits the protein function. Consequently, the fifth pathway may be a possible pathway for sengesterone (Figure 11(b)).

Based on the larger variation of protein structure, we suggest labiatic acid may have a stronger effect on *β*-CaMKII (Figure 12(a)). The possible pathway will be different for these interactions. After the calculation, the results indicate that these pathways move to similar positions until they project out of the pore (Figure 12(b)). This phenomenon indicates that labiatic acid not only determines the protein and pore variation, but also prevents interactions with other ligands.

Methyl 3-O-feruloylquinate causes variation in *β*-CaMKII by interacting closely with the amino acids and then the pore (Figure 13(a)). Although this pathway was predicted, we suggest, based on a 20 ns simulation, that this compound will occupy a position that will prevent other interactions and inhibit the protein function.

The results of structure variation indicate these compounds could cause protein variations that would result in inhibition of the protein function.

## 4. Conclusion

In the analysis of docking, the docking site and the ligand dock to the protein are correct based on the important amino acid interactions. The RMSD and energy show that *β*-CaMKII is a stable protein, according to low variation in the interaction and sample functional structure of important amino acids. *β*-CaMKII can maintain the composition of its structure during these important amino acid interactions. From this situation, we find that some proteins may prevent the large variations necessary for some amino acids to compose the functional domain. Finally, we suggest that these compounds may be able to regulate emotion, and we think sengesterone may have stronger effect than the others.

## Figures and Tables

**Table 1:** Screening the TCM database docking to CAMKII.

Name	Herb	-PLP1	-PLP2	Dock Score
Sengesterone	*Cyathula capitata* Moq.	75.71	69.85	134.220
Labiatic acid	*Rosmarinus officinalis* L.	84.03	87.55	111.237
Methyl 3-O-feruloylquinate	*Phellodendronchinense* C. K. Schneid.	44.46	45.03	109.576
Serpentine	*Catharanthus roseus* (L.) G. Don./*Rauwolfiabeddomei/Rauwolfia fruticosa/Rauwolfia serpentina *	70.29	60.45	102.18
Flazine	*Brucea javanica* (L.) Merr.	62.33	62.37	101.651
Xanthotoxol_8-O-beta_-D-glucopyranoside	*Glehnia littoralis* F. Schmidt *ex* Miq.	84.54	81.09	99.992
Ruine	*Peganum harmala* L.	80.96	76.7	97.767
3,3′,4-Tri-O-methyl ellagic acid	*Camptotheca acuminata* Decne.	77.81	66.18	95.091
(6aR_11aR)-9_10-Dimethoxypterocarpan-3-O-beta-D-glucoside	*Angelica sinensis* (Oliv.) Diels	106.26	105.61	93.609
Bixin	*Desmodium gangeticum* (L.) DC.	59.09	53.08	92.671

5CP_A_600∗		78.02	68.27	25.155

*Control.
